# Integrated molecular and behavioural data reveal deep circadian disruption in response to artificial light at night in male Great tits (*Parus major*)

**DOI:** 10.1038/s41598-022-05059-4

**Published:** 2022-01-28

**Authors:** Davide M. Dominoni, Maaike de Jong, Kees van Oers, Peter O’Shaughnessy, Gavin J. Blackburn, Els Atema, A. Christa Mateman, Pietro B. D’Amelio, Lisa Trost, Michelle Bellingham, Jessica Clark, Marcel E. Visser, Barbara Helm

**Affiliations:** 1 Institute of Biodiversity, Animal Health and Comparative Medicine, University of Glasgow, University Avenue, Glasgow, G12 8QQ UK; 2 Department of Animal Ecology, Netherlands Institute of Ecology (NIOO-KNAW), Wageningen, The Netherlands; 3 Plant Ecology and Nature Conservation Group, Wageningen University and Research, Wageningen, The Netherlands; 4 Glasgow Polyomics, Wolfson Wohl Cancer Research Centre, College of Medical, Veterinary and Life Sciences, University of Glasgow, Glasgow, G61 1BD UK; 5 Department of Behavioural Neurobiology, Max Planck Institute for Ornithology, Seewiesen, Germany; 6 FitzPatrick Institute of African Ornithology, University of Cape Town, Rondebosch, 7701 South Africa; 7 Centre d’Ecologie Functionnelle et Evolutive, University of Montpellier, CNRS, EPHE, IRD, Univ Paul-Valery Montpellier 3, Montpellier, France; 8 Groningen Institute of Evolutionary Life Sciences (GELIFES), University of Groningen, Nijenborgh 7, 9747 AG Groningen, The Netherlands

**Keywords:** Ecology, Physiology, Zoology

## Abstract

Globally increasing levels of artificial light at night (ALAN) are associated with shifting rhythms of behaviour in many wild species. However, it is unclear whether changes in behavioural timing are paralleled by consistent shifts in the molecular clock and its associated physiological pathways. Inconsistent shifts between behavioural and molecular rhythms, and between different tissues and physiological systems, disrupt the circadian system, which coordinates all major body functions. We therefore compared behavioural, transcriptional and metabolomic responses of captive great tits (*Parus major*) to three ALAN intensities or to dark nights, recording activity and sampling brain, liver, spleen and blood at mid-day and midnight. ALAN advanced wake-up time, and this shift was paralleled by advanced expression of the clock gene *BMAL1* in all tissues, suggesting close links between behaviour and clock gene expression across tissues. However, further analysis of gene expression and metabolites revealed that clock shifts were inconsistent across physiological systems. Untargeted metabolomic profiling showed that only 9.7% of the 755 analysed metabolites followed the behavioural shift. This high level of desynchronization indicates that ALAN disrupted the circadian system on a deep, easily overlooked level. Thus, circadian disruption could be a key mediator of health impacts of ALAN on wild animals.

## Introduction

On our rhythmic planet, organisms have adapted to the change of day and night by evolving circadian rhythms that are highly sensitive to light^1^. The near-ubiquity of circadian rhythms across kingdoms of life suggests major fitness benefits on two grounds. Internally, the circadian system regulates temporal coordination within the body to manage conflict and overlap between different processes. Externally, the circadian system anticipates environmental fluctuations, enabling organisms to align their behaviour and physiology with nature's cycles^1,2^. However, globally most humans and wild organisms in their vicinity are now exposed to artificial light at night (ALAN), and thus to a loss of the natural night^3^ that interferes with the refined functioning of the circadian system.

In animals, rhythmicity is primarily generated on a molecular level by a transcription-translation feed-back loop (TTFL), and is modulated by multiple interacting systems that involve neuronal, endocrine, metabolic and immune pathways. These processes are orchestrated by complex interactions between sensory input, central and peripheral clocks, and effector systems^4^, which are vulnerable to ALAN. There is ample evidence that activity rhythms are altered by ALAN, with possible consequences ranging from compromised human health to loss of ecosystem functions^5–8^. Yet, it is largely unclear to what extent ALAN disrupts animals at a deeper level: the circadian system could become desynchronized from activity patterns, and the physiological pathways it coordinates could become dis-integrated^9–13^. Thus, physiological changes associated with ALAN, including in endocrine, immune and metabolic pathways, might be caused or facilitated by circadian disruption^9,14–16^. Testing for such deep circadian disruption requires parallel behavioural and molecular approaches that examine effects of ALAN on activity and on different physiological pathways^8^.

Here we aim to fill this gap by an integrated, captive study of a bird, the great tit (*Parus major*), whose behavioural response to ALAN is well-characterized^12,17–20^. The birds' behaviour was closely monitored under a realistic range^21^ of experimental ALAN and in dark controls. We then measured gene transcripts in multiple tissues and blood metabolites using a day–night sampling design as in previous circadian disruption studies of humans^22,23^. The selected genes represented the circadian TTFL (Brain and Muscle ARNT-Like 1, *BMAL1*, alias *ARNTL*; cryptochrome 1, *CRY1*), a clock modulator (*casein kinase 1ε, CK1ε*)^24^, and endocrine, immune and metabolic pathways putatively affected by circadian disruption (Table S1). Tissues included central pacemaker and memory sites (hypothalamus, where important avian circadian pacemaker components are located^24^, and hippocampus; Fig. S1), and metabolic (liver) and immune tissues (spleen). Complementing the candidate gene approach, our untargeted metabolomics analysis captured both expected and novel effects of ALAN^25^. We aimed to identify whether (i) hypothalamic clock gene expression was affected by ALAN, (ii) potential temporal shifts in clock gene expression were consistent across tissues, (iii) behavioural and clock gene expression was aligned, and (iv) transcript and metabolite temporal shifts were consistent across physiological pathways. Any inconsistencies in temporal shifts indicate the potential for internal desynchronization, and hence, circadian disruption^8^.

**Figure 1:**
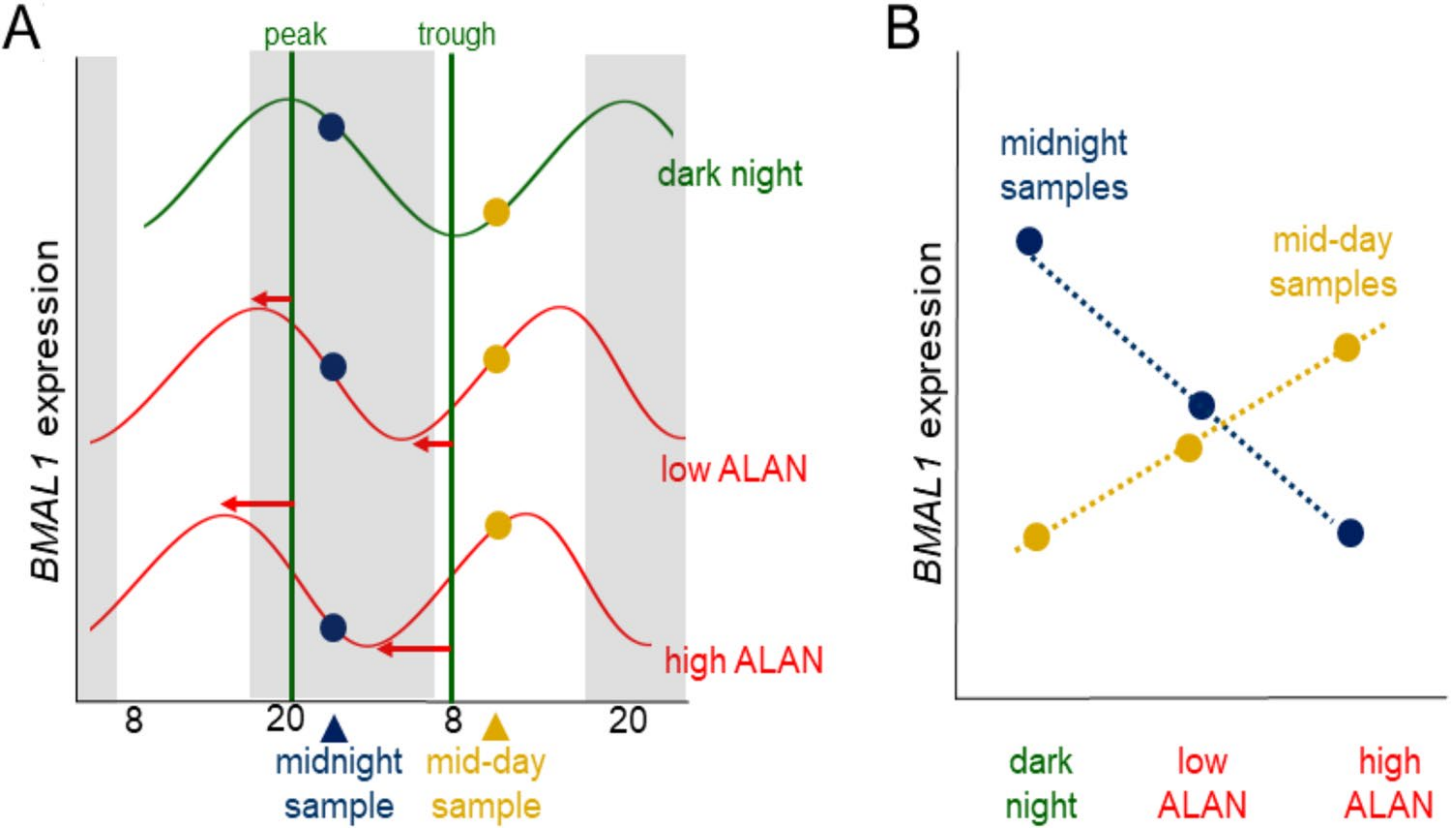
Expected clock gene rhythm advance in response to ALAN. Schematic shows ALAN effects on transcript levels of *BMAL1* measured at midnight (blue) and mid-day (yellow). (**A**) Rhythm of ALAN under dark night shown as green curve; if the gene's rhythm advances (red curves) with increasing ALAN, transcript levels sampled at midnight will drop, whereas those measured at mid-day will rise; horizontal arrows indicate the advance of the *BMAL1* peak. (**B**) The trends of transcripts with increasing ALAN therefore cross for mid-day vs. midnight sampling. This schematic focuses on effects on phase, assuming robust amplitude.

Our specific predictions are illustrated in Fig. 1, which shows expected patterns for *BMAL1*. Under dark nights (Fig. 1A, green curve), during midnight sampling (blue dots) *BMAL1* transcripts have just passed the peak (maximum), and during mid-day (yellow dots) they have just passed the trough (minimum). Under our hypothesis, the TTFL matches behaviour, and thus, with increasing ALAN (red curves), the *BMAL1* rhythm will also advance. Hence, at midnight *BMAL1* levels will be measured progressively later than the peak, and drop, whereas mid-day levels will be measured closer to the next peak, and hence rise. When combining midnight and mid-day data (Fig. 1B), we thus expected a cross-over of detected *BMAL1* levels. Other rhythmic compounds should show similar patterns, although the point of intersection and precise change of level depends on their phase. In contrast, if the TTFL remains unmoved by the behavioural shift by ALAN, compound levels will show as two horizontal lines across ALAN, representing day and night, respectively. Levels of non-rhythmic compounds will fall on a single line, representing both day and night.

## Results

### ALAN advances timing of activity and *BMAL1* expression

**Figure 2:**
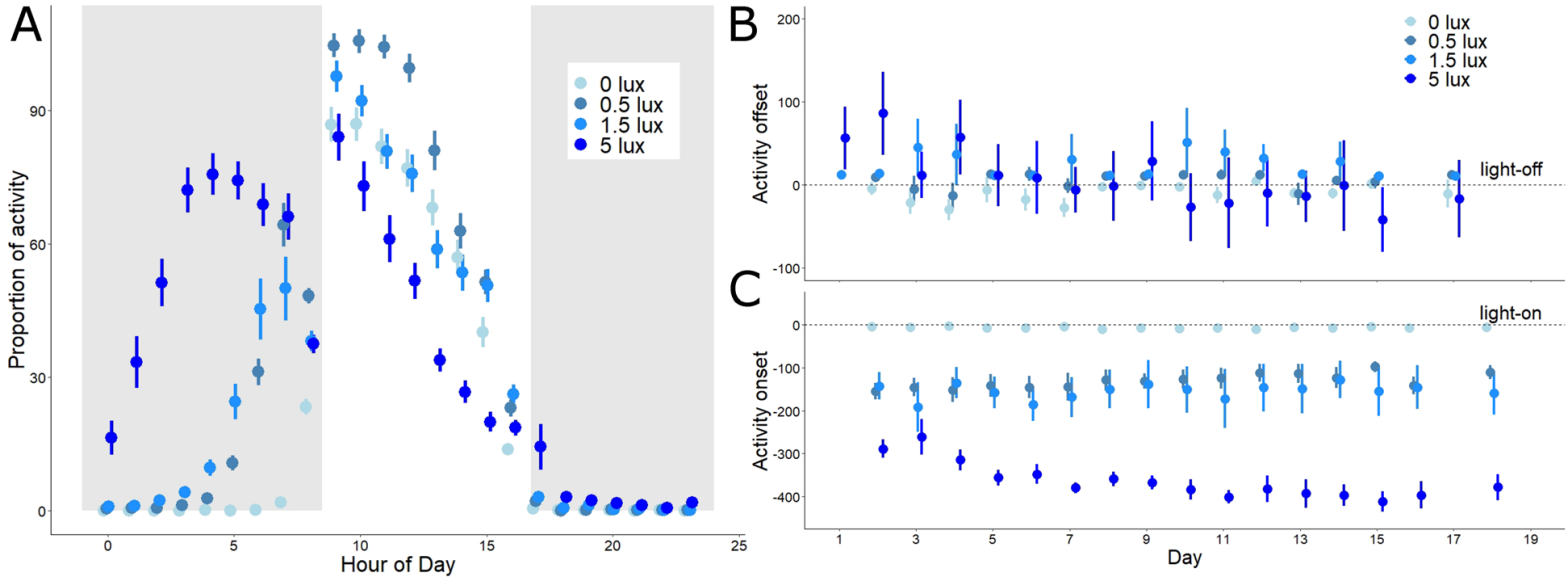
Activity timing is affected by intensity of ALAN. (**A**) Shows the proportion of active 2-min intervals in each treatment group per hour (raw mean ± SEM, N = 34). Grey background indicates night-time, white background indicates daytime. (**B**) and (**C**) show daily treatment group data (mean ± SEM), for the timing of evening offset and morning onset of activity, respectively (time in min). Activity onset and offset refer to times of lights-on and lights-off, which are shown as horizontal lines crossing zero.

Daily cycles of activity were strongly affected by the ALAN treatment (GAMM, p = 0.001, Fig. 2A and Fig. S2; Table S4). In the 5 lux group birds were generally active 6–7 h before lights-on, whereas birds in the other two light treatments (0.5 and 1.5 lux) advanced morning activity to a much lesser extent. Because of the advanced onset of activity, 40% of the overall diel activity in the 5 lux group occurred during the night, compared to 11 and 14% in the 0.5 and 1.5 lux groups, and less than 1% in the control dark group. Thus, with increasing ALAN, nocturnal activity also increased (LMM, treatment p < 0.001, Fig. 2A and Table S5).

Breaking down this average diel profile (Fig. 2A) by time since first exposure to ALAN (i.e., days from start of the experiment to first sampling, days 0 to 18) shows how the differences in activity timing developed (Fig. 2B,C). Upon exposure to ALAN, the birds’ activity onset (Fig. 2C) advanced in all treatment groups. The groups with intermediate light exposure (0.5 lux, 1.5 lux) responded by a similar instantaneous phase-advance (155 and 142 min, respectively, p > 0.1 for pairwise comparison), and thereafter their timing remained stable. The group exposed to 5 lux showed a much larger instantaneous phase advance of almost five hours (mean ± SEM = 289 ± 21 min), and thereafter continued to gradually phase-advance until reaching a stable phase after 10 days (interaction treatment × day, p < 0.001, Fig. 2C, Table S5). The advance until stabilization could represent gradual entrainment to an early phase. Equally, it could represent temporary free-run of activity under the reduced Zeitgeber amplitude (note that birds were not kept in constant light conditions), as suggested by periodogram analysis. Indeed, we found that in the 5 lux group, prior to stabilization, period length deviated from that of all other groups and from 24 h (mean period length 5 lux group: 23.6 h; LM; Table S6). The individual actograms (Fig. S3) further suggest that the activity rhythm in the 5 lux group may have split into an advancing morning component whereas evening activity remained largely stable.

Changes in the activity offset were much less pronounced (Fig. 2B). The 5 lux group showed an instantaneous delay phase-shift. This initial delay was followed by a gradual advance, similar to but smaller than that of morning onset. At the end of the experiment birds in the 5 lux group ceased their activity before lights-off, and earlier than other groups (treatment × day, p < 0.001, Fig. 2B, Table S5). This advance did not compensate for the earlier onset, as birds in the 5 lux group were more active over the whole 24 h than the remaining birds (treatment × day, p = 0.01, Table S5).

### Hypothalamic *BMAL1* expression at night parallels advanced activity onset

We next sought to identify whether the profound shifts in activity patterns were paralleled by corresponding shifts in the pacemaker, measured by expression of *BMAL1* in the hypothalamus. Day–night differences in transcripts of *BMAL1* inverted with increasing ALAN (Fig. S4A), as predicted above (Fig. 1). While *BMAL1* expression was higher at midnight than at mid-day for the control birds, increasing ALAN induced a reversal of this pattern, so that birds in the 5 lux group had much higher expression at mid-day than at midnight (treatment × time, p < 0.01, Table S7).

**Figure 3:**
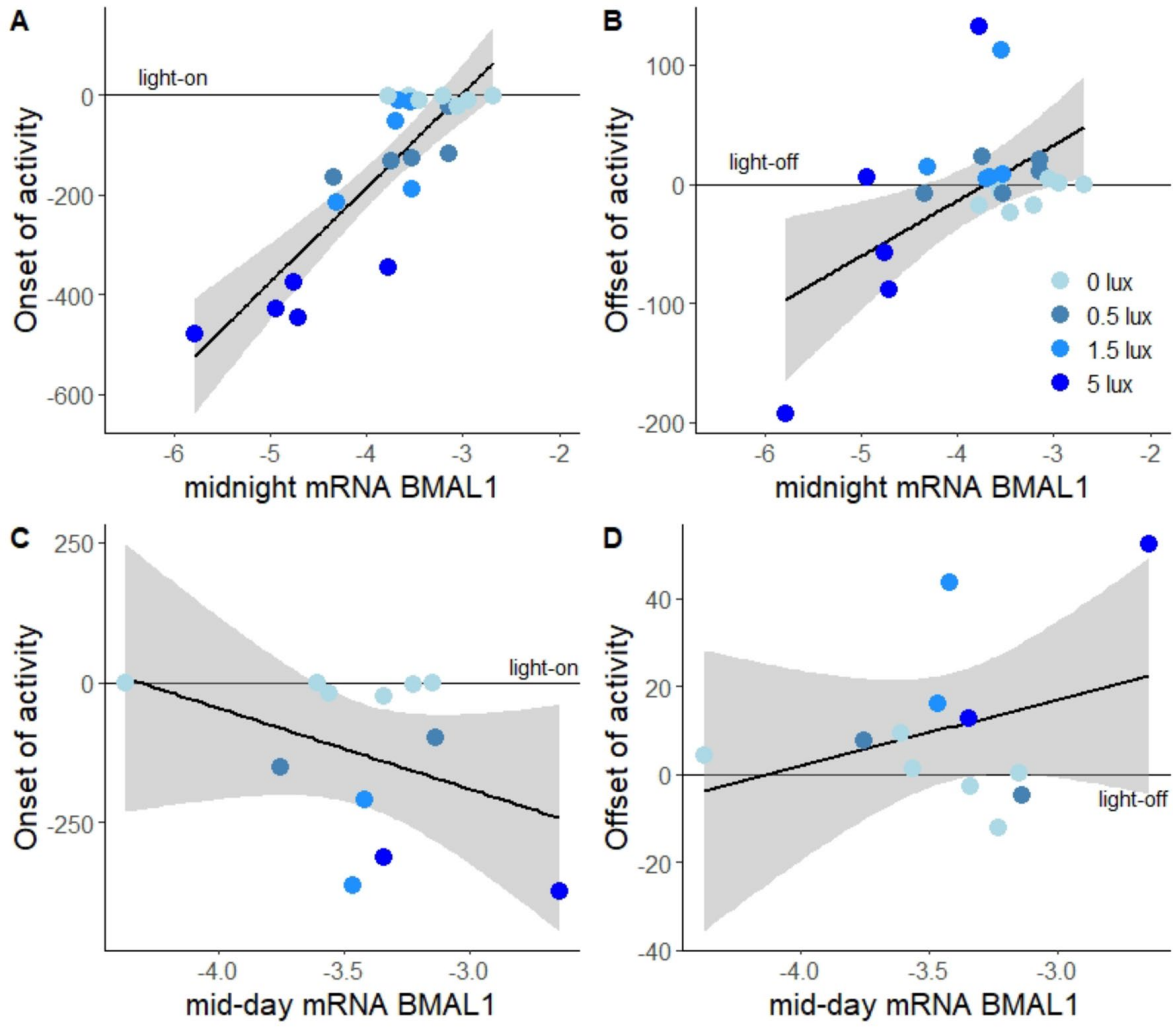
*BMAL1* expression in the hypothalamus predicts the advance of morning activity. mRNA levels of *BMAL1* at midnight correlated with the onset (**A**) and offset of activity (**B**), but mid-day levels did not (**C** and **D**). Shown are log-transformed mRNA levels, separated by ALAN treatments (blue colour gradient). Points represent individual birds, lines and shaded areas represent model fits ± 95% confidence intervals.

To assess whether changes in day–night *BMAL1* gene expression correlated with temporal behavioural shifts, we related *BMAL1* levels to onset of activity of an individual once it had stably shifted in response to the ALAN treatment (Fig. 2B,C, after 10 days). Onset was closely predicted by hypothalamic *BMAL1* expression at midnight (Gaussian LM, p < 0.001, R^2^ = 0.71, Fig. 3A). Across ALAN levels, the earliest rising birds had the lowest midnight expression of *BMAL1*. However, the steep linear regression was largely based on differences between ALAN groups in both activity timing (Figs. 2, 3) and *BMAL1* expression (Fig. S4A). Indeed, this relationship was even stronger when we only considered the 0.5, 1.5 and 5 lux group in the analysis (LM p < 0.001, R^2^ = 0.85), but the association was not present for the dark control birds (LM, p = 0.87). Individual midnight *BMAL1* levels also predicted mean offset of activity, albeit less strongly so than onset (LM, p = 0.006, R^2^ = 0.28, Fig. 3B). Conversely, mid-day *BMAL1* levels did not significantly predict variation in either activity traits (LMs, p > 0.1 and R^2^ < 0.16 for all measures).

### ALAN reverses day–night *BMAL1* expression patterns in multiple tissues

**Figure 4:**
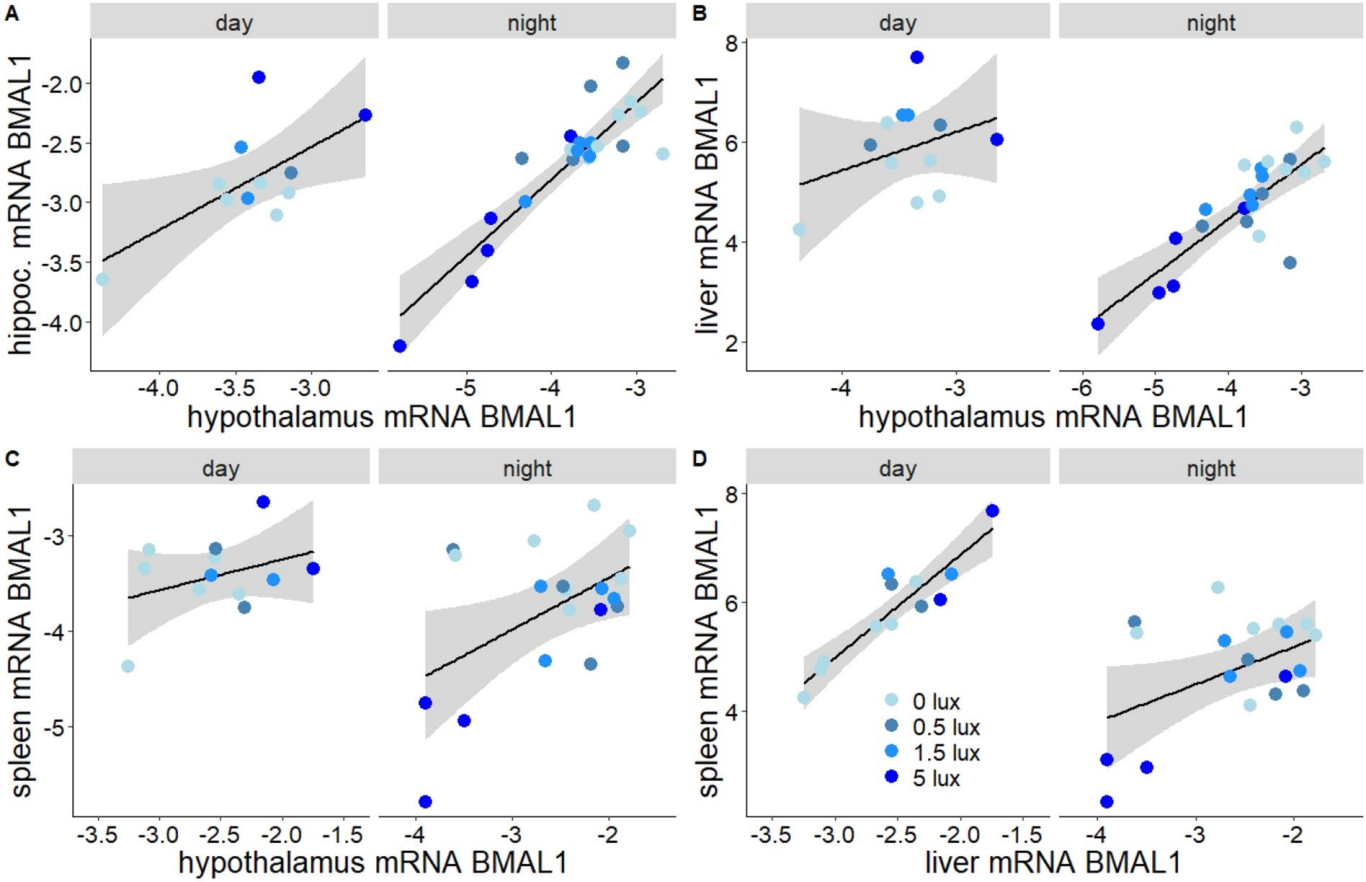
ALAN effects on *BMAL1* expression were comparable in different tissues. Correlation of expression patterns of *BMAL1* in different tissues. Shown are log-transformed mRNA levels, separated by sampling time (day vs night) and ALAN treatments (blue colour gradient). Points represent individual birds. Lines and shaded areas depict model estimated means ± 95% confidence intervals. Panels show expression levels of *BMAL1* in hypothalamus in relation to (**A**) hippocampus, (**B**) liver and (**C**) spleen, as well as spleen in relation to liver (**D**).

**Figure 5:**
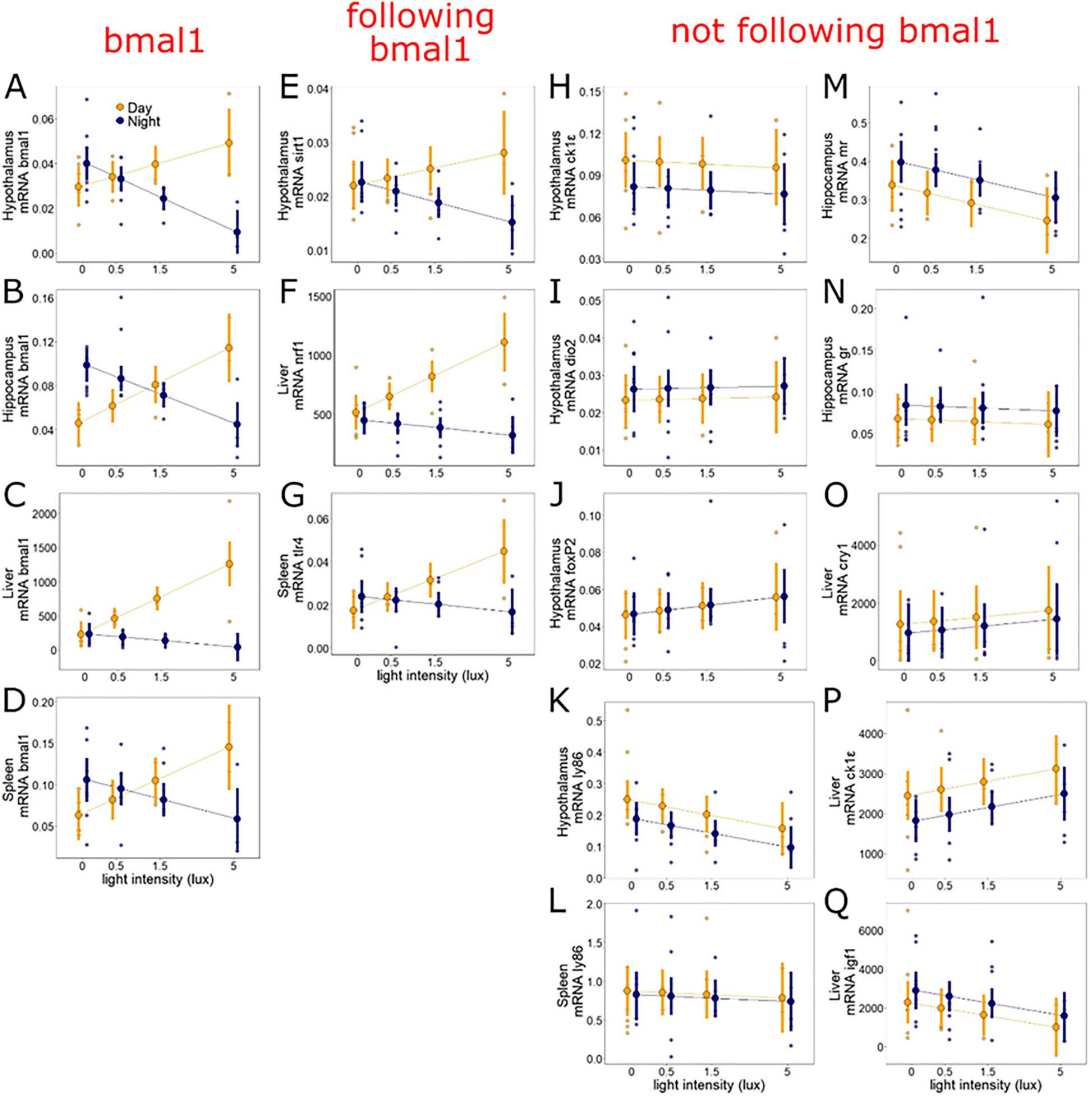
ALAN effects on gene expression are gene-specific. ALAN does not equally affect all physiological systems. ALAN effects on *BMAL1* (**A**–**D**) were paralleled by those on three additional genes in the hypothalamus (*SIRT1*), liver (*NRF1*) and spleen (*TLR4*) (**E**–**G**), but not by other genes analysed across tissues (**H**–**Q**). Shown are log-transformed mRNA levels, separated by sampling time (mid-day: yellow; midnight: dark blue). Large symbols ± SEM connected by lines represent model estimates, whereas small symbols depict raw data points (N = 34 birds).

ALAN-induced shifts in *BMAL1,* as detected in the hypothalamus, were remarkably consistent across tissues (Fig. 5A–D). Hippocampal *BMAL1* expression profiles resembled those in the hypothalamus (Fig. S5A; interaction of treatment and sampling time p < 0.001, Table S8). Within individuals, mid-day and midnight transcripts in both brain tissues were closely related (LM, p < 0.001, Fig. 4A, Table S9). Liver *BMAL1* showed similar effects of ALAN on day–night expression profiles (Fig. S6A; time × treatment, p < 0.001, Table S10), so that within individuals, hepatic and hypothalamic transcripts also correlated closely (LM, p < 0.001, Fig. 4B, Table S9). Similar ALAN effects on *BMAL1* expression were found also in the spleen (Fig. S7A; time × treatment, p = 0.003, Table S11), and individual-level transcripts closely correlated with those in hypothalamus (LM, p = 0.011, Fig. 4C) and liver (LM, p = 0.001, Fig. 4D, Table S9).

### Inconsistent shifts of expression patterns by ALAN in other genes

We then assessed whether the reversal of day–night expression patterns found for *BMAL1* was paralleled in other genes (Table S1). Our analysis revealed that different pathways were differentially affected by ALAN.

Among clock-related genes, hypothalamic expression levels of *CK1ε* were not affected by the light treatment (p = 0.71, Table S7). Expression was consistently, although not significantly, higher at mid-day (p = 0.09, Fig. 5H, Table S7). Expression of hepatic *CK1ε* slightly increased with light intensity (p = 0.078, Fig. 5P, Table S10), and was not affected by sampling time (p = 0.13, Table S10). In the liver *CRY1* showed no expression trend that aligned with that of *BMAL1* and was not affected by treatment or sampling time (p > 0.6 for both variables, Fig. 5O, Table S10).

Among metabolic genes, patterns similar to those in *BMAL1* were evident in *SIRT1*, a gene which is also involved in the modulation of the circadian cycle^26^. Hypothalamic *SIRT1* showed a clear change of day–night expression with increasing ALAN (Fig. 5E; treatment × time, p = 0.029, Table S7), and *SIRT1* levels were closely related to those of hypothalamic *BMAL1* (LM, p < 0.001, Table S9). In the liver, the metabolic gene *NRF1* showed a similar response to ALAN as *BMAL1*, with reversed day–night expression in the 5 lux group compared to other groups (treatment × time, p < 0.001, Fig. 5F, Table S10), and close correlation with *BMAL1* (LM, p < 0.001). In contrast, another hepatic metabolic gene, *IGF1,* was not significantly affected by light treatment or sampling time (for both, p > 0.11, Fig. 5Q, Table S10). In the hippocampus (Table S8), mid-day and midnight levels of the mineralocorticoid receptor, *MR*, decreased significantly with increasing ALAN (p = 0.044, Fig. 5M). Conversely, the levels of the glucocorticoid receptor, *GR*, showed no significant relationship with either light treatment or sampling time (p > 0.33 in both cases, Fig. 5N).

Among immune genes, hypothalamic *LY86* levels decreased with increasing ALAN (p = 0.04, Fig. 5K, Table S7), but the same gene in the spleen was not affected by either treatment or sampling time (p > 0.7, Fig. 5L, Table S11). Conversely, another immune gene in the spleen, *TLR4,* showed the same pattern as *BMAL1* (Fig. 5G, time × treatment, p = 0.006, Table S11).

Last, we also analysed genes involved in photoperiodic response in the avian brain. *FOXP2*, a gene that in birds is involved in learning, song development and photoperiod-dependent seasonal brain growth, showed no significant trends related to ALAN or sampling time (p > 0.32 in both cases, Fig. 5J). *DIO2,* a thyroid-axis gene involved in photoperiodic reproductive activation, was also not affected by either ALAN or sampling time (p > 0.45 for both variables, Fig. 5I).

### Metabolomic profiles support inconsistent reversal of day–night physiology under ALAN

**Figure 6:**
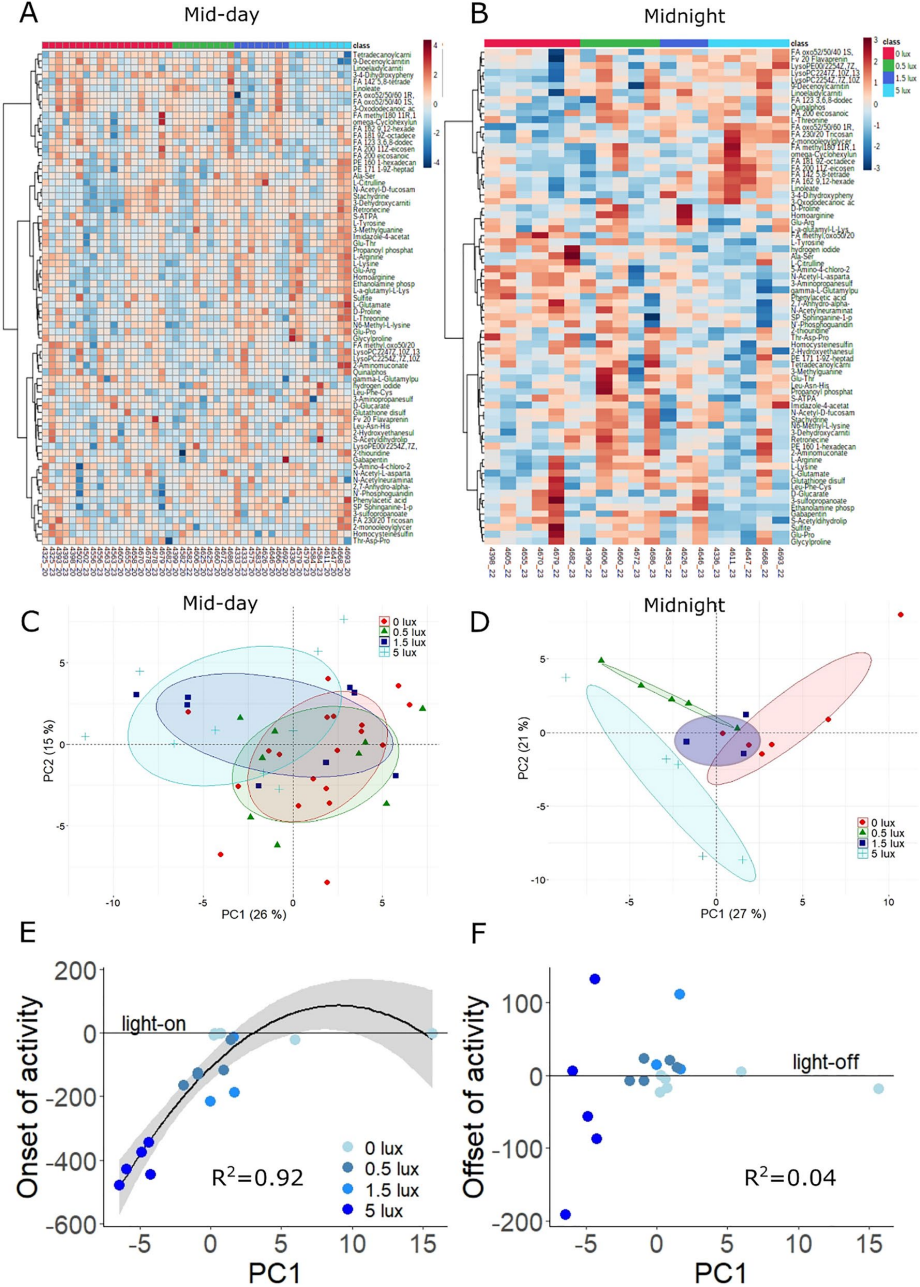
Metabolomics analysis supports ALAN-induced shifts in day–night physiology. The 73 metabolites found to be significantly affected by the interaction of treatment and sampling time (interactive dataset) were dissected by pathway analysis and PCA. Heatmaps show the top-25 metabolites at either mid-day (**A**) or mid-night (**B**). Heatmaps were created using the Metaboanalyst software^27^. PCA showed considerable overlap between ALAN groups at mid-day (**C**), whereas at midnight, ALAN treatment effects were pronounced, particularly for the 5 lux group (**D**). In all PCA plots, points represent individual samples, and ellipses contain 80% of samples in a group. The first PC of the night cluster significantly predicted the onset of activity (**E**), but not the offset of activity in the evening (**F**). In (**E**) and (**F**) points represent individual birds, and lines and shaded areas represent model fits ± 95% confidence intervals.

Untargeted LC–MS metabolomic analysis, after filtering, provided abundance values for 755 metabolites, which we tested for effects of ALAN and sampling time by individual linear mixed models (correcting for false discovery rate at 5%). We found that 44.1% (333) differed significantly by sampling time, with higher levels at mid-day in 197, and higher levels at midnight in 136 (all metabolite tables: 10.6084/m9.figshare.12927539.v1). 29 metabolites differed significantly by treatment (Table S12), whereby levels decreased with ALAN in 11 metabolites and increased in 18 metabolites. Finally, 73 (9.7%) of the 755 metabolites showed significant interaction between treatment and sampling time (Fig. 6 and Table S13; 34 of those also differed by sampling time). As this pattern supported reversal of day–night physiology similar to that shown for *BMAL1* expression, these metabolites were selected for subsequent focal analyses (hereafter named “interactive dataset”).

We dissected variation in the interactive dataset by using separate principal component analyses (PCA) on the samples collected at mid-day and midnight (Fig. 6C,D). For mid-day samples, ALAN treatments overlapped considerably (Fig. 6C), but some birds in the 1.5 lux and 5 lux treatments were separated by PC1 (26% of variance explained). PC1 in the mid-day dataset was heavily loaded with metabolites of Arginine biosynthesis pathway, including l-Arginine, Homoarginine and l-Glutamate, and with other important amino acids such as l-Threonine, l-Lysine and l-Tyrosine. Conversely, the midnight samples (Fig. 6D) separated clearly between the 5 lux treatment and the remaining groups. In this midnight PCA, PC1 explained 27% of the variance and was heavily loaded with metabolites of the Glutamate and Arginine pathways, as well as with *N*-acetyl-l-aspartate. PC2, which explained 21% of variation, was heavily loaded with fatty acids, including Linoleate (factor loading tables: 10.6084/m9.figshare.12927536.v1). The contribution of the Arginine pathway was further confirmed by pathway analysis, conducted with Metaboanalyst^27^, which indicated “Arginine biosynthesis” as highly significant (p < 0.001). “Aminoacyl-tRNA metabolism” (p < 0.001), “Histidine metabolism” (p = 0.005), and “Alanine, Aspartate and Glutamate metabolism” (p = 0.026) were also indicated as significant pathways.

We finally investigated whether, just like midnight levels of *BMAL1* expression (Fig. 4), midnight principal components of metabolites correlated with individual activity timing. PC1 strongly predicted the onset of activity via a linear and quadratic relationship (n = 19, p_linear_ = 0.007, p_quadratic_ = 0.014, R^2^ = 0.92, Fig. 6E), but did not explain offset of activity (p = 0.63, R^2^ = 0.04, Fig. 6F). PC2 was related to neither timing trait (p > 0.2).

## Discussion

Birds advanced the circadian timing of their activity with increasing levels of ALAN, corroborating earlier demonstrations of advanced daily activity under ALAN in captivity for several avian species, including the great tit^9,11,12,17^. In parallel the expression of our focal clock gene, *BMAL1,* was also advanced in the hypothalamus. Advances in *BMAL1* were consistent across tissues, indicating a shift of the circadian system in tissues implicated in timing, memory, metabolism and immune function. A similar molecular shift was observed in a comparison of clock gene expression in tree sparrows (*Passer montanus*) from an illuminated urban and dark non-urban habitat^28^. Sampled within a day of captivity, urban birds showed clearly advanced expression in clock genes, including, as in our birds, in hypothalamic *BMAL1*. Other avian studies have also reported effects of ALAN on rhythms in brain and other tissues^10,13^. Jointly, these data indicate that at least during exposure to ALAN, core elements of the circadian system shift alongside behaviour. Just how closely behaviour and clock elements are integrated is illustrated by our novel finding that activity onset at the individual level can be potently predicted by nocturnal expression levels in *BMAL1* (R^2^ = 0.71).

In apparent contrast to our findings, behavioural responses to ALAN were earlier interpreted as not involving the circadian clock^12^. In an experiment also on the great tit, Spoelstra and colleagues^12^ exposed birds to ALAN as in our study. Subsequently, birds were released to constant dim light (0.5 lux), where they showed free-running circadian activity rhythms. Intriguingly, the birds free-ran from the timing (phase) they had shown prior to exposure to ALAN, rather than from their advanced timing under ALAN. Thus, the study concluded that the birds responded to ALAN by behavioural masking, while the internal clock remained unchanged^12^. Our molecular data suggest otherwise, namely that within three weeks of ALAN exposure, internal time measured by *BMAL1* expression had phase-advanced in concert with behaviour. While these discrepancies are difficult to interpret because the studies differed in approach (molecular vs. behavioural inference) and in experimental design (inference from during ALAN vs. during ensuing free-run), our study gives clues to possible reasons. Firstly, on a behavioural level we observed some desynchronisation of rhythms under ALAN, in particular between activity onset and offset. Some birds in the 5 lux group (Fig. S3) displayed apparent splitting^29^ of activity into an advancing morning component and a more stably entrained evening component, suggesting internal desynchronization. Driven by the morning advance, the initial period length of the 5 lux group resembled the free-running period length of great tits from earlier studies under constant conditions^30^. Secondly, on a molecular level, advances in *BMAL1* were not paralleled by changes in a second clock gene we measured, *CRY1*, which might indicate that some features of the clock shifted while others ticked on.

Overall, in our study, only some of the investigated regulatory genes (Table S1) aligned with the ALAN-dependent advances of rhythms in behaviour and *BMAL1*. The genes from metabolic pathways that have close molecular links to the circadian TTFL, *SIRT1* and *NRF1*, mirrored ALAN-dependent changes in *BMAL1*. However, regulatory genes of immune pathways responded inconsistently, whereby *TLR4* aligned with *BMAL1* whereas *LY86* did not. The learning gene, *FOXP2* and the thyroid-activating gene *DIO2* did not mirror the changes in *BMAL1*, nor did the endocrine genes (*MR, GR, IGF1*). Conversely, in a complementary study on testes of these same birds, we observed that ALAN exposure, which also activated the reproductive system, shifted the day–night expression patterns of corticoid receptors^31^. Other experimental studies have confirmed that effects of ALAN on avian rhythms in brain and other tissues differed between genes and pathways^13^. For example, a study on zebra finches (*Taeniopygia guttata*) reported ALAN-induced changes in rhythmic expression of hypothalamic *CRY1* but not *BMAL1*^10^. This differs from our findings, and from findings that *BMAL1* and *CRY1*, but not another circadian gene, *CLOCK*, advanced in urban compared to rural tree sparrows^32^. However, core clock genes, as well as other genes that show circadian rhythms in expression, might differ in their phase and amplitude in different tissues^26,33^. Thus, while tissue-specific changes in gene expression due to ALAN may be expected, direct comparison between tissues could be misleading.

**Figure 7:**
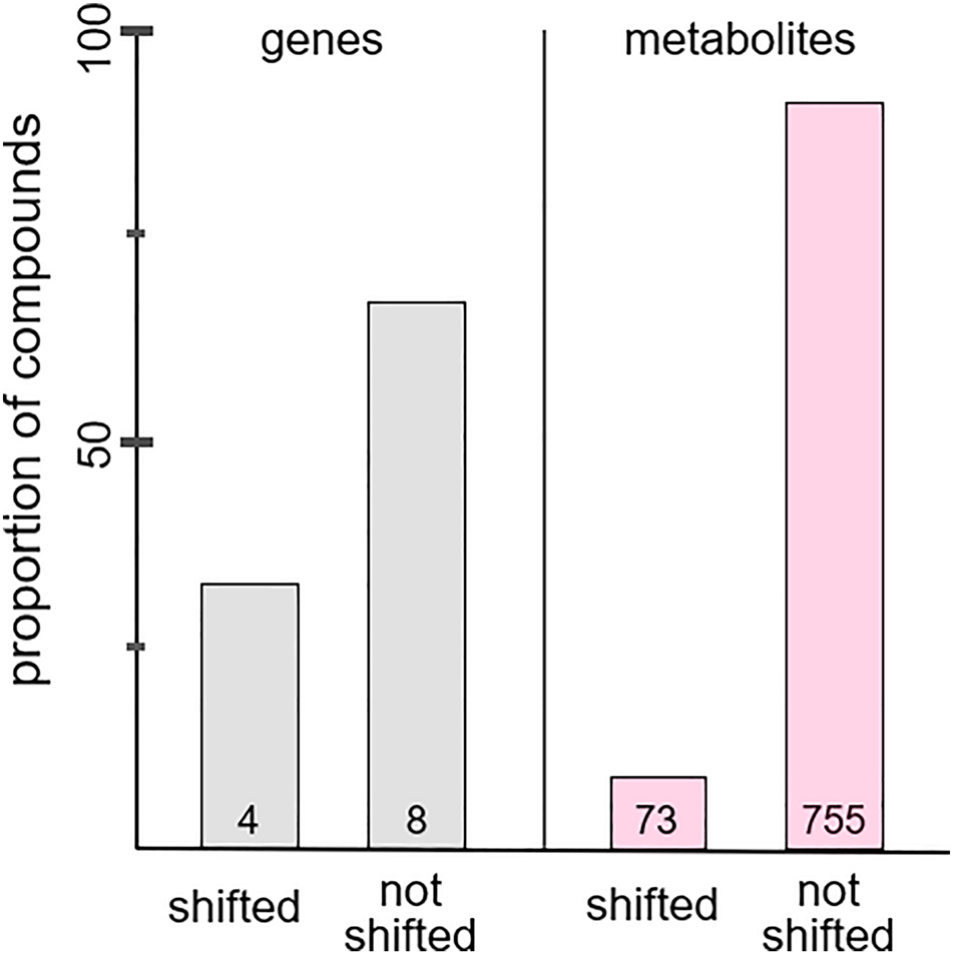
Proportion of shifts in day–night pattern in response to ALAN. Shown are proportions of genes (grey) and metabolites (red) whose levels were, or were not, significantly impacted by the interaction of sampling time and ALAN level.

Our metabolomic data corroborated dis-integrated shifts in physiology (Fig. 7). Of the 755 identified metabolites, nearly 50% (333) differed between mid-day and mid-night levels. However, less than 10% showed changes in rhythm under ALAN, although these select metabolites were powerful predictors of activity timing. Our findings that some, but not all, featured pathways aligned with shifts in behaviour and *BMAL1* converge with metabolomic studies of humans. To identify the mechanisms by which circadian disruption drives metabolic disorders and other pathologies, these studies severely disrupted the circadian system by sleep deprivation and shift-work protocols^25,34^. The reported changes in gene expression and metabolite levels were similar to those of our birds under ALAN. Both studies identified highly responsive pathways and compounds, in particular Arginine^25^, an amino acid strongly linked to circadian rhythms, innate immune responses^35^, and Glutamate production^36^. *N*-acetyl-aspartate, a metabolite linked to Glutamate^37^, was also observed to follow changes in behaviour and *BMAL1*. Because these compounds have diverse biochemical roles, further work would be required to determine which of these functions, if any, are associated with the behavioural and gene expression changes we observed. While preliminary, these data show the potential of metabolomic techniques for generating functional hypotheses for effects of ALAN on physiology.

Our study has several important limitations. First, the birds were used in a previous experiment which also manipulated ALAN levels^17^. In our experiment, we kept birds in similar light treatments as in the earlier experiment, as explained in the Methods (see Supporting Information for details), and the two experiments were interrupted by three weeks of dark nights for all birds. Thus, birds in our experiment had experienced pre-exposure to their respective ALAN treatments. After-effects of exposure to altered lighting schedules are long-known to have lasting effects on circadian rhythms^38^, for example depending on the amplitude and length of the previous photoperiod that an animal was exposed to^39,40^. Thus, we cannot exclude that previous exposure to ALAN might have affected the responses observed in this experiment. Second, the birds used in this experiment showed a strong, dose-dependent photoperiodic response to ALAN, as shown by faster testes growth and increased expression of genes linked to reproductive development^31^. The activation of the reproductive system is known to affect both exogenous and endogenous diel rhythmicity^29,41^. Thus, it is possible that some of the physiological and behavioural differences found between the treatment groups are an indirect consequence of increased reproductive activation, rather than a direct effect of exposure to ALAN. Confounded effects of ALAN on diel and annual clocks are well established^42^. However, for a detailed understanding of circadian disruption, future studies should aim at disentangling these two processes. We also concede that our study is based on a relatively low sample size and restricted to males for logistic reasons. Lastly, a caveat should be added regarding our day–night comparison of gene expression and metabolite levels. Day–night comparisons are an established tool for studying body time and offer insights into how the internal physiology may change in response to ALAN^22,23^. However, diel profiles based on several time points would have allowed a much deeper understanding of how ALAN affects circadian rhythms in physiology in different tissues.

Physiological effects of ALAN are becoming increasingly evident^6,7^. For example in captive birds, under ALAN molecular markers for sleep deprivation were elevated, hypothalamic expression of genes such as *TLR4* was altered^10^, neuronal features in the brain were changed, and cognitive processes and mental health-like states were impaired^10,15,43^. Altered hepatic expression of several metabolic genes further suggested negative effects on gluconeogenesis and cholesterol biosynthesis^9^. Free-living birds, including great tits, are also advancing daily activity under ALAN, although to a lesser extent than in captivity^44,45^, and often in onset but not offset^20,45^. Detrimental effects of ALAN on physiological pathways are beginning to become evident also in the wild^6,8^. Consequences of ALAN, such as ALAN-induced changes in immune function that increase host susceptibility to infectious disease^16^, may cascade from individuals to ecological or epidemiological scales. Addressing effects of ALAN is therefore urgent^6^.

Taken together, our study shows that birds shifted their internal clock time under ALAN, but suffered a high degree of internal desynchronization. Nonetheless, our data also indicate dose-dependent responses of behaviour and physiology^17,31,46^, which might allow mitigating against ALAN impacts on wildlife by reducing light intensity. Importantly, we detected substantial effects even at light intensities (0.5 lux) that are typically far exceeded by street illumination, and to which animals are exposed in the wild^21^. These findings transfer to other organisms including plants, insects, and mammals including humans^47–50^, and call for limits to the ever faster global increase in light pollution^3^.

## Methods

### Animals and experimental design

We studied 34 hand-raised, adult male great tits that were kept in individual cages (90 × 50 × 40 cm) under ambient temperature of 10–14 °C with ad libitum access to food and water, as described in^17^.

The experiment started on February 1st, 2014, when daylength was fixed at 8 h 15 min light and 15 h 45 min darkness. During the day, all birds were exposed to full spectrum daylight by high frequency fluorescent lights emitting ~ 1000 lux at perch level (Activa 172, Philips, Eindhoven, Netherlands). During the night, birds were assigned to four treatment groups exposed to nocturnal light intensity of 0 lux (n = 13), 0.5 lux (n = 7), 1.5 lux (n = 7), or 5 lux (n = 7) (Tables S2, S3) provided by warm white LED light (Philips, Eindhoven, Netherlands; for spectral composition, see^17^). Daily activity of each bird was measured continuously using micro-switches recorded by a computer, as described before^17^. See Supporting Information for definition of onset and offset of activity and for more details.

To derive tissues for molecular analysis, we sampled metabolites and transcripts at mid-day (3 h 30 min after lights on; i.e. 3.5 h Zeitgeber time) and midnight (7 h 15 min after lights off; i.e. 15.5 h Zeitgeber time). On Feb 20th an initial blood sample (~ 200 μl) was collected from all birds at mid-day for metabolomic profiling. On Feb 22nd birds were randomly assigned to mid-day or midnight groups for culling to collect tissues and blood (Table S2). The mid-day group was culled on Feb 22nd, whereas culling of the midnight group was divided over two nights (Feb 22nd: 12 birds; Feb 23rd: 10 birds). Organs were extracted, snap-frozen on dry ice, and stored at − 80 °C within 10 min of capture. Testes of the same birds were analysed in a separate study^31^.

All experimental procedures were approved by the Animal Experimentation Committee (DEC) of the Royal Netherlands Academy of Arts and Sciences and carried out under license “NIOO 13.11”. All methods were carried out in accordance with relevant guidelines and regulations. All methods are reported in accordance with ARRIVE guidelines (https://arriveguidelines.org) for the reporting of animal experiments.

### Gene expression analyses

Brain tissue was cut on a cryostat at − 20 °C into sagittal sections throughout the brain (Fig. S1). The hypothalamus and hippocampus were located by the use of the Zebrafinch atlas ZEBrA (Oregon Health and Science University, Portland, OR, USA; http://www.zebrafinchatlas.org) and isolated from the frozen brain sections either by surgical punches for the hypothalamus (Harris Uni-Core, 3.0 mm), or by scraping the relevant tissue with forceps, for the hippocampus. For the hypothalamus, the edge of the circular punch was positioned adjacent to the midline and ventral edge of the section, just above the optic chiasm, following the procedure of^51^. Hypothalamic and hippocampal tissue was immediately added to separate 1.5 ml buffer tubes provided by the Qiagen RNeasy micro extraction kit (see below), homogenized and stored at − 80 °C until extraction.

Whole spleens were homogenized with a ribolyser, and for livers, we cut and homogenized 0.5 g of tissue from each individual liver. RNA was extracted using the RNeasy micro extraction kit and reverse transcribed it to generate cDNA using a standard kit following the manufacturer’s instructions (Superscript III, Invitrogen).

Primers (for full details see Table S1) were built based on the great tit reference genome build 1.1^52^. RT-qPCR was performed on duplicate samples by a 5-point standard curve. We used reference gene levels to correct for variation in PCR efficiency between samples. The absolute amounts of the candidate genes were then normalized by division by the geometric mean of the absolute amounts of the reference genes. This step yielded relative mRNA expression levels of the candidate genes. See Supporting Information for further details.

### Metabolomics analysis

Following initial sample preparation, all samples (Table S3 for sample sizes) were analysed on a Thermo Scientific QExactive Orbitrap mass spectrometer running in positive/negative switching mode. The final peak set was filtered on the basis of signal to noise score, minimum intensity and minimum detections. Of a total of abundance values for 5483 compounds, 682 were annotated as known metabolites based on accurate mass and predicted retention time (40) and 73 were identified based on accurate mass measurement and matching retention time to a known standard (within 5%), resulting in a final dataset of 755 metabolites. See Supporting Information for more details.

### Statistical analysis

All statistical analyses were conducted in R, version 3.63^53^ (see Supporting Information for details). In all models we included treatment as log-transformed light intensity (adding a constant to avoid zero). To analyse locomotor activity data (i.e. perch-hopping), we divided the time series of activity into an unstable phase and stable phase (see Supporting Information). We used the data in the unstable phase to quantify circadian period length (tau) for each bird as in^12^, then tested treatment effects using a Gaussian linear model (LM). We analysed the data in the stable phase by first assessing how the treatment affected the proportion of time spent active every hour, using a generalized additive mixed model (GAMM). We then tested for treatment effects on onset time, offset time, nocturnal activity and total daily activity using linear mixed models (LMMs).

To examine variation in relative mRNA levels, we ran linear models (LMs) including ALAN treatment, sampling time (two-level factor, day and night), and their interaction as explanatory variables. Similar models were used to test for relationships in mRNA levels between the same gene in different tissues, or different genes in the same tissue, and between gene expression and locomotor activity traits.

We tested variation in the levels of the individual metabolites using all data, including the replicated mid-day samples, which correlated strongly within birds (Fig. S8) (total metabolomic samples: n = 64). We first ran independent LMMs for each metabolite, with metabolite levels as response variable (log transformed and normalized), and treatment, time of day and their interaction as explanatory variables. We then applied principal component analyses (PCA) to simultaneously analyse the 73 metabolites found to be significantly affected by the treatment × time interaction in the LMMs, run separately for samples collected at mid-day or midnight. Finally, we used the first two principal components (PC1 and PC2) of the midnight based PCA as explanatory variables in two LMs with onset and offset of activity as response variables, respectively.

## Supplementary Information

Supplementary Information.

## Data Availability

Raw data, created datasets and R scripts are available via Figshare: https://figshare.com/projects/Integrated_molecular_and_behavioural_data_reveal_deep_circadian_disruption_in_response_to_artificial_light_at_night_in_male_Great_tits_Parus_major_/88841.
